# Matrix Metalloproteinases and the Pathogenesis of Recurrent Corneal Erosions and Epithelial Basement Membrane Dystrophy

**DOI:** 10.3390/biology12091263

**Published:** 2023-09-21

**Authors:** Katarzyna Jadczyk-Sorek, Wojciech Garczorz, Beata Bubała-Stachowicz, Tomasz Francuz, Ewa Mrukwa-Kominek

**Affiliations:** 1 Department of Ophthalmology, University Clinical Center, Medical University of Silesia, Ceglana 35, 40-514 Katowice, Poland; 2 Department of Ophthalmology, Faculty of Medical Sciences in Katowice, Medical University of Silesia, Ceglana 35, 40-514 Katowice, Poland; 3 Department of Biochemistry, Faculty of Medical Sciences in Katowice, Medical University of Silesia, Medyków 18, 40-027 Katowice, Poland

**Keywords:** matrix metalloproteinases, epithelial basement membrane dystrophy, Cogan’s microcystic dystrophy, recurrent corneal erosions

## Abstract

**Simple Summary:**

Based on our previous studies on the levels of selected matrix metalloproteinases (MMPs) in patients with recurrent corneal erosions (RCE), we made a detailed assessment of their possible contribution to the development of corneal epithelial basement membrane dystrophy. The existing literature describing the structure, nomenclature, activation, and substrate specificity of metalloproteinases, as well as factors affecting their activity, are summarized. A separate section focuses on the effect of metalloproteinases on the corneal healing process, which is a preview of the final considerations on the effect of metalloproteinases on the development of recurrent corneal erosions and corneal epithelial basement membrane dystrophy. Our previous experimental studies revealed elevated metalloproteinase concentrations in the corneal epithelium of patients with recurrent corneal erosions concomitant with epithelial basement membrane dystrophy. These MMP concentrations are correlated with histopathology and confocal microscopy findings typical of this group of patients. Based on the consistency of the obtained results, the authors suggest a contribution of matrix metalloproteinases to the development of corneal epithelial basement membrane dystrophy.

**Abstract:**

Matrix metalloproteinases (MMPs) are a group of proteolytic enzymes which are members of the zinc endopeptidase family. They have the ability to degrade extracellular matrix elements, allowing for the release of binding molecules and cell migration. Although metalloproteinases regulate numerous physiological processes within the cornea, overexpression of metalloproteinase genes and an imbalance between the levels of metalloproteinases and their inhibitors can contribute to the inhibition of repair processes, the development of inflammation and excessive cellular proliferation. The involvement of MMPs in the pathogenesis of dystrophic corneal diseases needs clarification. Our analyses focus on the involvement of individual metalloproteinases in the pathogenesis of recurrent corneal erosions and highlight their impact on the development of corneal epithelial basement membrane dystrophy (EBMD). We hypothesize that abnormalities observed in patients with EBMD may result from the accumulation and activation of metalloproteinases in the basal layers of the corneal epithelium, leading to basement membrane degradation. A barrier formed from degradation materials inhibits the normal migration of epithelial cells to the superficial layers, which contributes to the development of the aforementioned lesions. This hypothesis seems to be lent support by the elevated concentrations of metalloproteinases in the corneal epithelium of these patients found in our previous studies on the relationships between MMPs and recurrent corneal erosions.

## 1. Introduction

Matrix metalloproteinases (MMPs) are a group of proteolytic enzymes which are members of the zinc endopeptidase family. MMPs were discovered in 1962 during studies on tadpole metamorphosis by Jerome Gross and Charles M. Lapiere, who described an enzyme that degrades the triple helix of collagen, later named metalloproteinase 1 (MMP-1) [1]. The name “matrix metalloproteinases” was not applied until the 1989 scientific conference in Sandestin Beach, USA [2,3], where the nomenclature of MMPs was adopted [4].

MMPs are produced by various cell types, including connective tissue cells, vascular endothelial cells, leukocytes, and macrophages. Epigenetic processes [5,6], gender [7], and age [8,9] appear to play an important role in MMP expression. Their activity is regulated both at the level of gene expression and by activation from inactive proenzymes. Gene expression can be inhibited by transforming growth factor beta (TGF β) and glucocorticoids. Regulation at the level of enzymatic activity involves MMP activation from pro metalloproteinases. MMP activity inhibition is mediated by tissue inhibitors of matrix metalloproteinases (TIMP) and non-specific plasma inhibitors [10,11,12,13,14]. Another and less well-described level of MMP regulation involves the controlled absorption/elimination of active proteases from the extracellular environment [15].

As proteinases, MMPs have the ability to degrade the elements of extracellular matrix (ECM), i.e., collagen, fibronectin, proteoglycans and laminin, allowing for the release of binding molecules and enabling cell migration. MMPs are also involved in the formation of biologically active protein fragments, activation and deactivation of other proteinases, synthesis and release of receptors for hormones, cell adhesion molecules, signaling molecules, and growth factors. Thus, they participate in the transmission of signals into the cell and control cell proliferation, migration, differentiation, and adhesion [5,10,11,12,16,17]. Under physiological conditions, such activity is vital in the processes of angiogenesis and embryonic development. In pathology, dysregulation of MMPs and TIMPs leads to inflammatory conditions, autoimmune diseases, abnormal angiogenesis, fibrosis, and carcinogenesis [7]. In particular, the imbalance between MMPs and TIMPs has been implicated in lifestyle diseases, such as diabetes, its vascular complications, and nephropathy. Also, the sequelae of cardiovascular disease, especially vascular remodeling, depend on metalloproteinases. To date, no therapy based on MMP inhibition has been registered for clinical use in chronic diseases. The specificity of the inhibitors tested was not limited to a single MMP, and systemic inhibition may not be beneficial [7,15].

## 2. Matrix Metalloproteinases—Structure, Nomenclature, Activation, and Inhibition

### 2.1. MMPs—Structure and Nomenclature

MMPs are multi-domain enzymes composed of at least one catalytic domain and a prodomain (except for MMP-23). In addition, the most common components of MMPs are the hemopexin domain and the region connecting it to the catalytic domain [5,11,18,19].

The catalytic domain is responsible for the proteolytic activity of MMPs. It contains a catalytic center containing one catalytic and one structural zinc ion, and usually three calcium ions. The active site is located on the enzyme’s surface in a groove that divides the domain into two subunits: smaller and larger. This site determines the substrate specificity [11,19].

The prodomain contains a propeptide that keeps MMPs in an inactive form (zymogen proenzyme, proMMP). It consists of three α-helices connected by flexible loops. The fourth ligand in the zymogen is the cysteine SH group, which interacts with the catalytic zinc ion, keeping proMMPs inactive. Upon activation, the connection between cysteine and Zn^2+^ is disrupted allowing the enzyme to bind to the target ligand. The structures of the MMP-1, MMP-2, MMP-3, and MMP-9 prodomains have been described previously [11,19].

The hemopexin domain enables MMP-9 to bind the tissue inhibitor of metalloproteinases [11] and is essential for the activation of proMMP-2 [20,21]. According to some authors, it is also responsible for stabilizing the enzymes [11,21]. MMP-12 sheds the hemopexin domain upon its activation while MMP-7, MMP-23, and MMP-26 do not have it at all [11,19,22,23].

**Figure 1:**
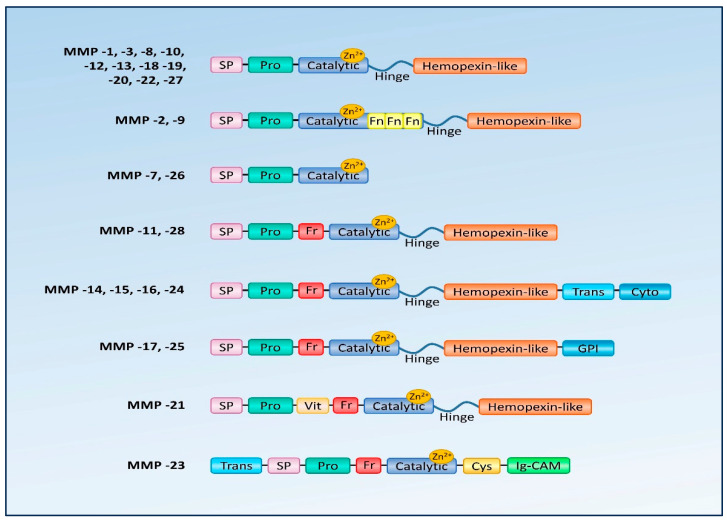
Domain structure of matrix metalloproteinases. SP—signal peptide; Pro—propeptide; Catalytic—active catalytic domain; Hinge—linker domain; Hemopexin-like—hemopexin like domain; GPI—glycosylphosphatidylinositol anchoring domain; Fr—furin site; Fn—fibronectin like domain; Vit—vitreonectin domain; Trans—transmembrane segment; Cyto—cytoplasmic tail; Cys—cystein rich segment; Ig-CAM—immunoglobulin-like cell adhesion molecule domain.

The linker region connecting the catalytic domain to the hemopexin domain is responsible for maintaining the stable structure of the enzyme molecule but also appears to be important in the degradation of some metalloproteinase substrates, such as collagen [11,24]. Mutations of this region in MMP-1 and MMP-8 markedly reduce their collagenolytic activity [19]. The structure of the matrix metalloproteinase domains is shown in Figure 1.

To date, 28 different metalloproteinases have been described, differing in three-dimensional (3D) structure and substrate specificity. Molecules referred to as MMP-4, MMP-5, MMP-6, and MMP-22 are not on the list because their structure and functions are identical to other MMPs [4,19]. The original MMP classification is based on domain organization and substrate preference; hence several MMPs groups have been described, i.e., collagenases (MMP-1, -8, -13, -18), gelatinases (MMP-2, -9), stromelysins (MMP-3, -10, -11), and a heterogeneous group with matrilysins (MMP-7, -26), epilysin (MMP-28), and membrane-type MMPs (MMP-14, -15, -16, -17, -24, -25) [11,19]. Seven of the MMPs have not been classified into any of these groups, although the structure of MMP-12, MMP-20, and MMP-27 is similar to that of stromelysins. X-ray crystallography and NMR spectroscopy allowed for the determination of 3D structures of MMPs, which led to a broader understanding of enzyme action and an alternative classification of MMPs according to their domain structure [19]. MMPs and their substrates are described in Table 1.

### 2.2. MMP Activation

MMPs are produced as inactive proenzymes. The prodomain restricts substrate entry and catalysis in the catalytic pocket by blocking the catalytic zinc ion through cysteine attachment [11,19]. MMPs become activated via the removal of the prodomain, thereby exposing the enzyme’s active site. Activation can occur due to the action of active MMPs (MMP-1, -7, -13), other proteinases (plasmin, thrombin), some specific environmental parameters (elevated temperature, low pH), chemical agents such as mercury compounds, oxidized glutathione or denaturing surfactants [5,11]. Some of the MMPs, which contain a specific sequence located at the terminal end of the C-prodomain, i.e., the furin recognition sequence (KX(R/K)R), are activated intracellularly in the Golgi apparatus. These include MMP-1, all membrane MMPs, and MMP-21 [11]. Activation can also occur at the cell surface with the participation of membrane-type matrix metalloproteinases (MT-MMPs). Among others, proMMP-2 is activated in this way [11,21]. 

### 2.3. MMP Inhibitors

Under in vivo conditions, MMP activity inhibition is mediated by tissue-specific inhibitors (TIMPs) and non-specific plasma inhibitors, such as α2-macroglobulin or α1- antiproteinase [10,11,12,13,14].

To date, four tissue MMP inhibitors have been described: TIMP-1, TIMP-2, TIMP-3, and TIMP-4. TIMP-1 and TIMP-3 are glycoproteins while TIMP-2 and TIMP-4 do not contain a carbohydrate group. They differ in their expression, which can be constitutive or induced and are regulated at the transcriptional level by various cytokines and growth factors. Their distribution in tissues also differs [13]. TIMP-1 has the lowest inhibitory potential and low affinity for MT-MMP, MMP-14, MMP-16, MMP-19, and MMP-24, while TIMP-3 has the broadest spectrum of action and is tightly bound to the extracellular matrix, which distinguishes it from the others [25]. Despite the apparent trend, some deviations from the general pattern have been noted, e.g., TIMP-2 and TIMP-3 have lower affinity for MMP-3 and -7 compared to TIMP-1 [14]. 

In addition to inhibiting proteolytic enzyme activity, TIMPs have been shown to have anti-angiogenic, pro- and anti-apoptotic effects [14]. It has also been reported that cell signalling mechanisms mediated by TIMP-1 and -2 promote cellular proliferation [14,26].

In normal corneas, TIMP-1 was found mainly in epithelial and endothelial cells; much lower amounts of TIMP-1 were noted in corneal stroma keratocytes. TIMP-2 and TIMP-3 were found in both epithelium, stromal keratocytes, and corneal endothelium. In corneas of pseudophakic eyes with bullous keratopathy, the pattern of TIMP-1 was similar to normal corneas. Regarding TIMP-2, its amount in epithelial cells, keratocytes, and endothelial cells of normal corneas and corneas with bullous keratopathy and eyes after radial keratectomy remained at similar levels. TIMP-3 has been reported in epithelial cells, keratocytes, and endothelial cells of normal corneas [27]. 

Due to the multifaceted involvement of MMPs in disease processes, MMP inhibitors are considered potential candidates for treating conditions such as cancer, neurodegenerative diseases, cardiovascular diseases, and inflammatory diseases. They could also have essential roles in treating ophthalmic conditions. 

## 3. The Process of Corneal Healing—Involvement of MMPs 

Corneal wound healing is a multidimensional process with four phases: hemostasis, inflammation, cell proliferation, and tissue remodeling [28]. The healing response is usually caused by epithelial or endothelial injury, which can also involve the stroma. However, it can also be triggered by immune or infectious processes that enter the stroma through the limbal blood vessels [29]. The complex interactions during corneal healing are mediated by autocrine, juxtacrine, and paracrine pathways and involve the corneal epithelium, stromal keratocytes, the nerves (cranial nerves V and VII), the lacrimal gland, and the tear film [30,31,32]. Cytokines and growth factors (GF) that regulate interactions include epidermal growth factor (EGF), hepatocyte growth factor (HGF), fibroblast growth factor (FGF), platelet-derived growth factor (PDGF), keratinocyte growth factor (KGF), nerve growth factor (NGF), and insulin-like growth factors (IGF-1, IGF-2), as well as transforming growth factor-beta (TGF-β) and tumor necrosis factor-alpha (TNF-α) [29,31]. In the intact cornea, the cytokines mentioned above and GFs are constitutively expressed and stored intracellularly by the corneal epithelium, corneal nerves, keratocytes, and corneal endothelium. Essential for cell growth and functionality, they help maintain corneal homeostasis; some are secreted in a limited and controlled manner, mainly in a latent or inactive form [31,32,33].

Apoptosis of keratocytes is the earliest event observed in the stroma after epithelial damage and remains a likely target for the modulation of the overall response that occurs in wound healing [30,32,33,34]. It has been shown that the more extensive the damage to the corneal epithelium and underlying stroma, the higher the level of keratocyte apoptosis [33]. 

Several studies indicate that interleukin-1 (IL-1) is a key regulator of processes involved in the corneal healing cascade [29,30,32,33,35,36]. Both IL-1α and IL-1β are produced constitutively in the corneal epithelium [29,35,37]. The IL-1 receptor (which binds both IL-1α and IL-1β) is also constitutively expressed in keratocytes [38,39,40]. IL-1α and IL-1β are not present in keratocytes of the intact cornea, nor has IL-1 been shown to be released from the epithelium into the corneal stroma [29,30,34,41]. However, it has been noted that exposure to IL-1 can induce its production by keratocytes through an autocrine loop [42,43]. IL-1 is released from cells when they are damaged or undergo apoptosis as part of the physiological process of epithelial renewal or necrosis associated with damage to the ocular surface, so it may be present in tears [29,44]. As long as the corneal epithelial structure remains intact, IL-1 has no ability to enter the anterior corneal stroma. Once the epithelium is damaged, IL-1 enters the stroma, where it can bind to IL-1 receptors on keratocyte cells, thereby having the ability to modulate their function [30,32]. IL-1 has been shown to both affect keratocyte apoptosis and activate keratocytes to produce HGF and KGF [32,34,36,37], which mediate epithelial and corneal stroma interactions to regulate the proliferation, differentiation, and apoptosis of the healing epithelial cells [29,36,45].

It has been shown that the proliferation of keratocytes begins about 12–24 h after injury and persists for at least ten days [29,41,46]. During this time, cytokines and chemokines released by injured epithelial cells, as well as chemokines produced by keratocytes in response to IL-1, attract bone marrow-derived cells, such as monocytes, macrophages, lymphocytes, and fibrocytes, to the corneal stroma. Many of these cells also produce chemokines and cytokines that enhance the response [29,30,47]. As a result, apoptotic bodies are removed, and the area affected by remodeling is cleared. If corneal damage is linked to tissue invasion by microorganisms, inflammatory cells eliminate pathogens [30]. The process of repair and restoration of corneal homeostasis can continue for several months or even years after injury.

Soon after corneal epithelial damage, there is also an increase in the expression of growth factors in the lacrimal glands, such as the aforementioned HGF and EGF, which accelerate the healing of the corneal epithelium [48]. The increase in tear secretion occurs via a reflex loop, through the sensory endings of the trigeminal nerve (V cranial nerve), which, connecting through the brainstem, generates an impulse transmitted via the facial nerve (VII cranial nerve) to the lacrimal gland [29,49]. 

In parallel with the release of IL-1α and IL-1β by damaged corneal epithelial cells, TGFβ1, TGFβ2, and PDGF are up-regulated, released, and activated by the corneal epithelium and matrix and enter the corneal stroma [50]. In a normal, undamaged cornea, epithelial production and activation of TGFβ and PDGF are relatively low. After injury, their accumulation in the stroma triggers the transition of keratocytes to fibroblasts and further development to mature myofibroblasts [29,50]. The latter produce high levels of collagen, hyaluronan, and biglycan, creating a disorganized and opaque cornea, clinically observed as corneal haze in the anterior stroma. Under physiological conditions, myofibroblasts undergo apoptosis after corneal repair when the epithelial basement membrane (EBM) is completely regenerated [51]. In corneal injuries that heal correctly, corneal fibroblasts begin but do not complete their development into mature myofibroblasts. This process may take months or years or never occur if corneal scarring (fibrosis) is permanent [29]. 

Expression of matrix metalloproteinases was reported in human corneal tissues in the 1990s [52]. Specific structural organization of collagen and the continuous restoration of normal corneal tissue architecture through dynamic intercellular transformations regulated mainly by MMPs allow for corneal transparency [16]. For this reason, the cornea is an illustrative experimental model for studying this group of enzymes [28]. 

During corneal healing, MMPs and TIMPs can be released, contributing to matrix remodeling by removing irregular matrix and restoring newer ECM [51]. Furthermore, since MMP substrates include cytokines, cell adhesion molecules, and active matrix components, proteolytic modification of these substrates by MMPs can affect cell signaling and tissue patterning. Thus, MMPs can act as essential regulators of cellular activity and serve as a link between cells and ECM [16]. MMPs have been shown to activate signaling molecules such as TNFα through MMP-3 [53] or TGF-*β* through MMP-9 [54]. MMPs also have the ability to cleave cell adhesion molecules, such as E-cadherin [55], galectin-3 [56] and L-selectin [57]. Changes in adhesion status enable cell migration and tissue integrity. 

In the process of corneal healing, MMPs may be produced through rapid or long-term transformations [16].

The first mechanism is characterized by a rapid peak in MMP-9 activity during epithelial wound healing [58]. The rapid corneal regeneration pathway concerns re-epithelialization, that is, the reconstruction of the corneal epithelium. The essential function of all epithelia is to act as a barrier between the external environment and the body’s tissues. In the case of injury, re-epithelialization begins immediately after tissue damage. It involves the restoration of the EBM and cell migration. It has been shown that the leading edge of the newly formed epithelium consists of a single layer of flattened cells. Actin filaments are anchored by cadherin and coordinate the movement of the progressing epithelial margin [29,59]. Once the epithelial defect is closed, the basal epithelial cells initiate EBM regeneration by producing self-assembling laminins, which then trigger the formation of mature EBM consisting of other laminins, perlecan, nidogens, and type IV collagen [60]. For repair processes to proceed correctly, intercellular junctions and those between cells and ECM components are degraded, allowing epithelial cells to adopt a migratory phenotype, which is mainly made possible by enzymes from the MMP group [16]. Data show that MMP-9 is the first to be synthesized and secreted by corneal epithelial basal cells at the edge of the migrating epithelium after injury, thus controlling the resynthesis of the EBM by stimulating cells to migrate. MMP-9 activity begins up to 24 h after injury and declines over several weeks when the basement membrane of the corneal epithelium is fully restored [58]. Fini et al. showed that overexpression of MMP-9 leads to impaired re-epithelialization and causes chronic corneal ulceration, usually observed after thermal injury. Inhibition of MMP activity in this model leads to improved basement membrane integrity [61].

When the process of re-epithelialization and restoration of EBM is completed, the developing myofibroblast precursors are removed, and corneal transparency is maintained or can be restored [29,34]. If EBM is not regenerated, myofibroblasts mature and transform into scar tissue. Fibrotic scarring will persist until normal EBM is restored; until then, corneal clarity is impaired [29,50,51,60]. It has been observed that even minor injuries, such as corneal abrasion, can induce corneal fibrosis due to recurrent epithelial defects and unfinished EBM regeneration [62]. Singh et al. emphasize that an imbalance between MMPs and TIMPs in favor of TIMPs can promote fibrosis, leading to tissue remodeling, as in the case of MMP-9 [17].

Long-term transformation involves MMP secretion by resident stromal fibroblasts and slow remodeling of the wound area. Expression of MMP-1, MMP-3 [63], actin [64], and integrin α5 [65] facilitates fibroblast repair and collagen reticulum restoration. As mentioned above, the primary signaling molecule in collagen restoration is Il-1α. Importantly, IL-1 has been shown to increase the secretion of metalloproteinases and collagenases by keratocytes via an autocrine IL-1 loop [42,43,58,66,67]. It has also been indicated that the IL-1 receptor antagonist expressed by corneal epithelial cells downregulates MMP-2 produced by corneal fibroblasts [68]. Moreover, Il-1 potentiates the PDGF on corneal fibroblasts. PDGF has been reported to be expressed in corneal epithelial cells. Analogous to Il-1, damage to the epithelium and underlying basement membrane results in PDGF release into the stroma, where it stimulates corneal fibroblasts to proliferate and differentiate [69,70,71]. PDGF is also secreted by neutrophils; MMPs synthesis occurs in regions of high neutrophil infiltration in the healing cornea [61]. 

Several studies revealed that the level of MMP-1, MMP-2, and MMP-3 rose for several weeks after injury and reached a maximum with the peak in fibroblast accumulation in the repaired tissue [58,61,72]. Mulholland et al. [73] evaluated the effect of MMPs on corneal regeneration after anterior keratectomy (AK) and lamellar keratectomy (LK). They found that after the AK wound, MMP-1 was a key mediator of epithelial migration, while MMP-2 and MMP-9, and to a lesser extent MMP-3, might participate in the remodeling of corneal stroma and the reformation of EBM. In contrast, an LK wound resulted in a much lower stimulus for MMP activation. It has also been suggested that the action of MMP-2 in stromal repair is partly independent of epithelial injury.

Some authors have also highlighted the role of MMP-7 [74], MMP-13, and MMP-14 [75] in the corneal healing process. Lu et al. [74] showed that basal epithelial cells secrete MMP-7 during the migration-proliferation phase of corneal wound healing after excimer keratectomy, while Ye et al. [75] demonstrated that the expression of MMP-14 and MMP-13 in animal corneas is comparable to that of MMP-2 and MMP-9, respectively. MMP-13 may play an essential role in the proteolytic cascade associated with MMP-9, which enables rapid turnover of ECM components during corneal wound healing. MMP-14 may be involved in removing abnormal ECM components by activating MMP-9 in rat corneas. 

Detailed analyses of aspects of corneal tissue modeling and repair mechanisms after injury and burns (including evaluation of the role of MMPs in these processes) are aimed at identifying targets for new therapies. An important evolving aspect is the use of nanotechnology in delivering drugs that currently have low bioavailability. It has been shown that nanocarriers help deliver anti-inflammatory drugs through the corneal epithelial barrier to act directly at the site of damage [76]. Thus, re-epithelialization is promoted, and corneal fibrosis/scar formation is prevented.

## 4. The Role of MMPs in the Pathogenesis of Recurrent Corneal Erosions and Epithelial Basement Membrane Dystrophy 

Although MMPs regulate numerous physiological processes within the cornea, including the aspects of tissue remodeling and regeneration outlined above, the literature emphasizes their involvement in abnormal corneal healing after burns [12,77,78] or trauma [79,80]. Overexpression of MMP genes and an imbalance between the levels of MMPs and their inhibitors can contribute to the inhibition of repair processes, the development of corneal inflammation, ulceration [12,77,81,82,83,84,85,86], and excessive and abnormal cellular proliferation [85,87,88]. 

MMPs involvement in the pathogenesis of dystrophic diseases is an interesting yet still not fully explored area. The following analyses focus on the involvement of individual MMPs in the pathogenesis of recurrent corneal erosions and highlight their impact on the development of epithelial basement membrane dystrophy.

### 4.1. Epithelial Basement Membrane Dystrophy—Epidemiology, Clinical Manifestations, and Molecular Background

Epithelial basement membrane dystrophy (EBMD), also referred to as Cogan’s microcystic dystrophy, anterior basement membrane dystrophy, or map-dot-fingerprint dystrophy [89], occurs in 2–6% [90] of the general population and is estimated to be more common in women [91]. Symptomatic disease mainly develops in patients over 50 [92]. EBMD is classified as a dystrophic disease—an inherited disorder that affects, either singly or in combination, cells, tissues, and/or organs; however, it does not have a well-documented heredity and may have a degenerative or secondary to trauma etiology [92]. Only one publication identifies two families with EBMD with *TGFBI* mutations [92]. The main symptoms are recurrent corneal epithelial erosions, monocular diplopia, “ghost images”, and a loss of visual acuity, although EBMD may also be asymptomatic [92,93,94,95].

**Figure 2:**
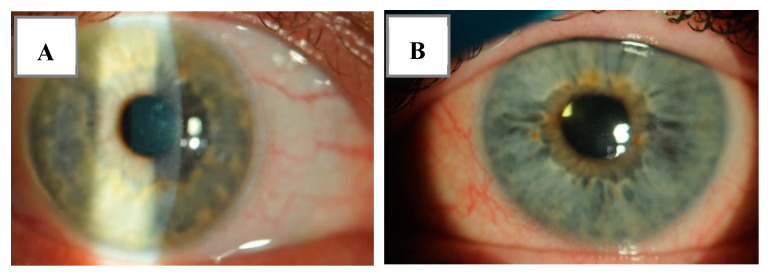
(**A**,**B**). Photograph of a patient with corneal epithelial basement membrane dystrophy.

Histologically, EBMD is characterized by intraepithelial microcysts, basal cells invaginating into the corneal epithelium, and irregular subepithelial accumulation of fibrogranular material, all of which form an image of maps, dots, blebs, and fingerprint-like patterns visible during slit-lamp examination [92,96] (Figure 2A,B).

**Figure 3:**
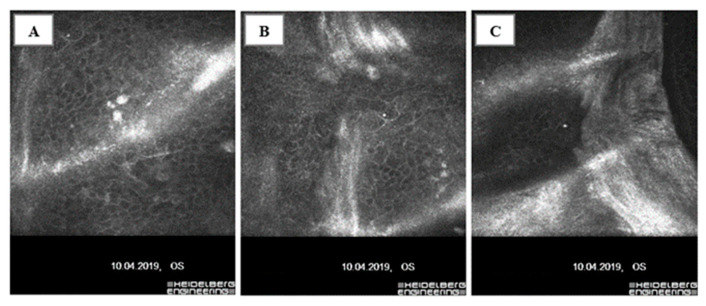
(**A**–**C**) Confocal microscopy scans of a patient with corneal epithelial basement membrane dystrophy.

The histological findings are consistent with confocal microscopy images [97] (Figure 3A–C).

### 4.2. Recurrent Corneal Erosions—Etiology, Epidemiology, and Pathogenesis

Recurrent corneal erosions were first described in the ophthalmic literature by E. Hansen in 1872 as “intermittent neuralgic vascular keratitis” [98], but it was Chandler who correctly categorized this disease entity [99,100].

The majority of recurrent corneal erosions (45–64%) are the consequence of traumatic lesions, e.g., acute, sudden abrasion of the corneal epithelium. The second most common cause of recurrent corneal erosions is EBMD, which accounts for 19–29% of the cases [20,99,101,102]. Less commonly, RCEs occur in the course of other dystrophies involving the corneal epithelium (Messmann dystrophy, subepithelial mucinous corneal dystrophy), Bowman’s membrane (Reis-Bucklers, Thiel-Behnke and Grayson-Wilbrandt corneal dystrophy), and the stroma (granular, lattice and macular corneal dystrophy), as well as in endothelial dystrophies (Fuchs dystrophy) accompanied by corneal decompensation and the formation of bullous keratopathy [99,103,104,105].

RCEs may also accompany ophthalmic conditions such as severe dry eye syndrome, corneal stem cell deficiency, meibomian gland dysfunction, anatomical abnormalities of the eyelids causing lagophthalmos [106,107] or may occur as a consequence of surgical procedures, such as corneal refractive surgery or corneal transplantation [99]. Systemic endocrine, metabolic, or autoimmune disorders may also influence the course of the disease. Increased susceptibility to the disease has been shown in people with rosacea [108] and diabetes [109,110]. 

The highest prevalence has been reported in the third and fourth decades of life [105,111]. However, in clinical practice, there are two peaks in the prevalence of RCEs, depending on the cause of the erosion. The first peak occurs in younger patients around 30 with post-traumatic RCEs, and the second peak concerns older patients around 50 with RCEs due to corneal dystrophy. These observations are consistent with results obtained in experimental studies [112]. There are no data on the variability of the prevalence by gender or ethnicity [97,105,111,113]. 

Since recurrent corneal erosions are characterized by periodic spontaneous incidents of corneal epithelial damage, their pathogenesis involves a defect in anchoring the corneal epithelium to the basement membrane. Normal adhesion and stability of the cells of the epithelial basal layer are maintained due to hemidesmosomes and the ordered structure of the corneal epithelial basement membrane, made up of plectin and integrin α6β4, which binds to laminin, an extracellular matrix protein. In addition, the lamina densa of the basement membrane is composed of collagen (types IV, XIII, XVII), laminin, perlecan, and nidogens. Anchoring fibers between the lamina densa and Bowman’s membrane made of type VII collagen provide stability [97,114].

Previous theories on the causes of recurrent erosion have focused on microstructural abnormalities in the interface between the epithelium and basement membrane. The instability of the corneal epithelium at the surface mainly results from a deficit of hemidesmosomes or an abnormal structure of the corneal EBM [97,115]. 

In addition to microstructural deficits, biochemical interactions between components of the epithelial-stromal complex are also important. Current research focuses on the role of neuropeptides, which regulate cell migration, proliferation, and differentiation and facilitate cell adhesion [105]. Substance P and IGF-1 have been shown to participate in the healing of RCEs and the restoration of normal corneal cytoarchitectonics [116]. NGF and glial cell-derived neurotrophic factor also contribute to corneal nerve regeneration and wound healing [117]. However, enzymes from the metalloproteinases group, which exhibit degradative effects against components of the epithelial anchoring complex to the basement membrane, appear to play a paramount role; they are believed to be the leading cause of successive recurrences of corneal erosion [118,119,120]; Table 1 provides a detailed list of MMPs and their substrates.

### 4.3. MMPs and the Pathogenesis of Recurrent Corneal Erosions 

Evaluation of MMP-2 concentrations in the corneal epithelium of patients with RCEs showed significantly higher MMP-2 concentrations compared to healthy individuals. Interestingly, a comparative analysis of patients with traumatic RCE and EBMD showed that MMP-2 levels were higher in those with EBMD. The differences in MMP-2 concentrations between the groups were not statistically significant, but the trend towards higher concentrations in the group of patients with EBMD was noticeable [112]. 

In a study evaluating the activity of MMP-2 and MMP-9 gelatinases in corneal epithelium, MMP-2 expression was shown to be increased in epithelia affected by recurrent erosion compared to samples from healthy individuals [119]. Based on analysis of their activity in tears, it was proposed that increased gelatinase activity in the affected eye during the remission phase and in the unaffected fellow eye may contribute to the recurrence of corneal erosion [120]. Interestingly, neither the active form of MMP-2 nor that of MMP-9 was detected in the samples from traumatic corneal erosion patients and control individuals [120], possibly due to the fact that MMPs can be significantly diluted in tears in states of excessive secretion, causing a false negative result. The corneal epithelium appears to be a more stable environment for MMP testing.

Based on studies evaluating the effect of MMP-2 on corneal regeneration after injury, it was found that the presence of MMP-2 correlates positively with the period of migration and activation of keratocytes at the wound site and that it is mainly found behind the leading edge of the migrating epithelium which may indicate its role in the long-term remodeling of the stroma and the restoration of the basement membrane of the corneal epithelium [16,58,73,121].

Analyzing the effect of MMP-2 on corneal epithelial dysfunction after thermal burn in animal models, Fini et al. showed that the lack of re-epithelialization correlated with an increase in gelatinases (including MMP-2) recorded in the corneal stroma of the rat on day 1 after injury at the site of fibroblast inflow to the injured area. Its level continued to rise for several weeks after injury and reached a maximum with a peak in fibroblast accumulation in the repaired tissue. These data support the concept that the overexpression of matrix metalloproteinases by resident corneal cells compromises re-epithelialization after certain types of corneal injury [61].

Similar analyses apply to MMP-3, found in the healthy corneal epithelium; however, its mean concentration is much lower than that of MMP-2. Notably, significantly higher concentrations of MMP-3 have been reported in the corneal epithelium of RCE patients compared to the healthy population. When analyzed by primary cause of RCE, higher MMP-3 concentrations were noted in patients with epithelial basement membrane dystrophy and lower in patients with post-traumatic RCE. The differences in MMP-3 concentrations between the groups were not significant. Thus, the distribution of results between groups was similar to those obtained for MMP-2; however, the values of MMP-3 concentrations were significantly lower [112].

Based on studies of MMP activity in the corneal stroma after injury, it has been shown that MMP-3 has transient and weak activity in the corneal stroma; its correlation with migration and activation of keratocytes at the wound site, basement membrane synthesis, and stroma remodeling is lower than that of MMP-2 and MMP-9 [16,58,73,121,122]. In corneal injury, the expression of MMP-3 (like that of MMP-1 and MMP-2) becomes pronounced in the stroma and increases gradually over several months after injury. MMP-3 overexpression was noted in the corneal stroma on day 1 after injury, at the site of accumulation of fibroblasts migrating to the area of injury. Its levels rose steadily over several weeks after injury, reaching a maximum value proportional to the maximum number of fibroblasts in the repaired tissue [61]. 

The findings regarding MMP-9 activity in the corneal epithelium of patients with recurrent corneal erosions are somewhat different. MMP-9 has not yet been demonstrated in the healthy corneal epithelium [119,122]. However, some authors have shown its activity in patients with RCE [119,120]. The timing of the collection of material for the study is crucial. The growth dynamics of MMP-9 after injury are different from the other MMPs involved in corneal tissue regeneration. MMP-9 appears up to 24 h after injury. The peak of its maximum concentration at the site of injury occurs earlier than that of the other MMPs, is associated with re-epithelialization, and declines within a few weeks after injury when the basement membrane of the corneal epithelium is fully restored [58,61,122]. Our study evaluating the levels of MMPs in corneal epithelium from RCE patients collected during phototherapeutic keratectomy, i.e., in a period of disease remission, showed no evidence of MMP-9 in the samples tested [112]. The reason could be that the tissue was collected after the erosion had healed, suggesting that although MMP-9 is the first to be synthesized and secreted by corneal epithelial basal cells at the edge of the migrating epithelium after injury, thus controlling the resynthesis of the epithelial basement membrane by stimulating cells to migrate [58,61,73,122], its concentration level has little or no effect on the development of subsequent erosions [112]. This appears to be an important issue, as some authors [118,123] recommend the systemic use of tetracycline, an MMP-9 inhibitor, to prevent recurrent corneal erosions. While its administration in the acute phase of the disease, when MMP-9 levels remain high, is fully justified, its prophylactic use during the remission phase is questionable.

### 4.4. The Role of MMPs in the Pathogenesis of Epithelial Basement Membrane Dystrophy 

Early analyses of the involvement of MMPs in the development of RCEs have focused on patients with post-traumatic etiologies [119,120], making it possible to analyze the contribution of MMPs to corneal healing after injury.

Based on our previous studies of MMP concentrations in the corneal epithelium of RCE patients [112], which detail the precise etiology of RCEs, comparative analysis of MMP-2 and MMP-3 concentrations in EBMD and traumatic RCEs, showed a trend towards higher concentrations in the former, which is an essential observation regarding the pathogenesis of this disease entity. The significantly higher concentrations of selected MMPs in the corneal epithelium of all patients compared to healthy individuals and the trend of higher MMP concentrations in EBMD compared to post-traumatic RCEs can be interpreted from a broader perspective. Such analysis may also provide some insight into the pathogenesis of EBMD. 

As mentioned before, the etiology of EBMD is not precisely defined; it is suspected that it may have a degenerative background. Therefore, our investigations in the field are fully justified, and further research is well worth pursuing.

If it is assumed that, in EBMD, epithelial instability on the surface results primarily from hemidesmosome insufficiency or a genetically determined pathology of the basement membrane, MMPs concentrations should not be higher than those of traumatic RCE patients. Since the basement membrane is unaffected by chronic degeneration in the latter, MMPs could be a leading cause of surface corneal epithelial instability. The perspective changes with an assumption that in EBMD, the excessive activation of MMPs is responsible for the abnormal structure of the basement membrane, as this would trigger a vicious circle mechanism. Excessive amounts of MMPs localized in the basal layer of the corneal epithelium cause damage to the components of the epithelial-stromal complex, leading to the accumulation of degraded material that forms a barrier preventing the proper differentiation and migration of epithelial cells from the basal to the more superficial layers, resulting in their damage or apoptosis in the inner layers. Excessive accumulation of degraded epithelial cells activates MMPs to degrade them through Il-1 release and activation. And so, the vicious circle closes. As described in Section 3, with the corneal epithelial structure intact, IL-1 is unable to enter the anterior corneal stroma. After epithelial damage, IL-1 enters the stroma, where it can bind to IL-1 receptors on keratocyte cells, stimulating them to secrete MMPs and other collagenases. IL-1 also intensifies the chemotactic effects of PDGF on corneal fibroblasts [30,44]. PDGF is also secreted by neutrophils, and it should be noted that MMP synthesis takes place in regions of high neutrophil infiltration into the healing cornea [61]. 

The above mechanism would explain the epithelial and basement membrane structure changes in EBMD shown in histopathological specimens [93,96,124]. Under normal conditions, the continuous proliferation of peri-stromal epithelial basal cells gives rise to new layers, which then differentiate into superficial cells. As these cells mature, they become covered with microvilli on their outer surface, then exfoliate. As previous analyses have shown, the cause of the epithelial microcysts characteristic of EMBD is the so-called midepithelial lamina, which is composed of irregularly striated fibers embedded in a granular matrix and is a product of basement membrane degradation. Microcysts develop posterior to this lamina as it blocks forward migration of epithelial cells. Entrapped cells undergo modifications in situ that adapt them to their normal role as superficial cells. This is followed by apoptosis, autolysis, and subsequent seclusion as microcysts. Microcysts are not formed via phagocytosis. Cyst walls are formed by adjacent cells that flatten and surround the dying cells without engulfing them. The presence of microcysts in the epithelium’s more superficial layers may result from their migration through lacunae in the midepithelial lamina [124].

So far, no clear answers have been obtained regarding how the midepithelial lamina is formed. However, intercellular and surface accumulation of acid mucopolysaccharides (glycosaminoglycans) has been shown to be an early sign of cell disruption that precedes microcyst formation [96]. Perhaps the above MMPs substrate degradation products cause the formation of an intermediate epithelial layer and thus block its migration toward the surface, affecting the formation of microcysts. In addition, the theory of MMP involvement in the pathogenesis of corneal EBMD could be supported by the fine lines characteristic of EBMD, also located at the midepithelial layer. They are multilayered structures; the superficial layer contains granular material, and the deeper layer is composed of banded fibrils, probably abnormal type IV collagen. The latter is a component of corneal epithelial basement membrane and an MMP substrate. It can be concluded that the damage occurs due to excessive accumulation of MMPs in this area [96]. 

## 5. Conclusions

The literature highlights EBMD as one of the leading causes of RCEs, in which increased MMP activity has been demonstrated. However, the effect of MMPs on the development of EBMD has not been directly analyzed to date. Furthermore, previous analyses evaluating the impact of MMPs on RCEs were mainly based on studies in patients with post-traumatic RCEs. Our findings with respect to MMP levels in the corneal epithelium of patients with both traumatic RCEs and EBMD allowed us to put forward a hypothesis regarding the effect of MMPs on the development of EBMD.

The above analyses indicate that deposits in the basal cells of the corneal epithelium and folds, streaks, and microcysts under the basal layer of the corneal epithelium and superficial corneal plexus nerves of patients with corneal epithelial basement membrane dystrophy observed in histopathological specimens and by confocal microscopy may result from MMP accumulation [93,96,97,124]. Such accumulation occurs in the basal layers of the corneal epithelium. It leads to the destruction of the basement membrane of the corneal epithelium and the formation of a barrier from its degradation products that inhibits the normal migration of epithelial cells into the superficial layers. This hypothesis seems to be supported by the elevated levels of MMPs in the corneal epithelium of EBMD patients demonstrated during studies on the effects of MMPs on RCE [112].

Further research in this area is certainly warranted. At present, many issues remain unexplained. An important aspect that remains open for further research is determining TIMP levels in the corneal epithelium and the relationship between MMPs and TIMPs in this disease entity. As shown by works on the involvement of MMPs in other corneal diseases, the interplay of enzymes and their inhibitors is crucial to maintaining the balance between ECM synthesis and degradation, which remains a prerequisite for preserving the structural and functional integrity of the cornea. Certainly, the evaluation of MMP inducers like the EMMPRIN protein or the evaluation of Il-1 levels as an activating factor for fibroblasts to secrete MMPs in corneal epithelial damage is also worth considering. 

## Figures and Tables

**Table 1:** MMPs and their substrates.

MMP	Common Name	Analogous Structure or Substrate Affinity with Other MMPs	Selected Degradable MMP Substrates
MMP-1	collagenase	MMP-8, -13, -18	collagen type I, II, III, V, VII, VIII, IX, X, XI
MMP-2	gelatinase A	MMP-9	collagen type I, IV, V, VII, X, gelatin, elastin
MMP-3	stromelysin 1, proteoglycanase	MMP-10, -11	elastin, proteoglycans, aggrecans, gelatin, proMMP-1, proMMP-8, proMMP-9, collagen type III, IV, V, IX, IX
MMP-7	matrilysin 1, metalloendopeptidase	MMP-26	collagen type IV, gelatin glycoproteins
MMP-8	collagenase 2	MMP-1, -13, -18	collagen type I, II, III, IV
MMP-9	gelatinase B	MMP-2	collagen type I, II, III, IV, XI, XVI, fibronectin, gelatin, laminin, osteopontin
MMP-10	stromelysin 2	MMP-3, -11	collagen type I, II, III, V
MMP-11	stromelysin 3	MMP-3, -10	laminin, antitrypsin
MMP-12	elastase, MME	-	elastin
MMP-13	collagenase 3	MMP- 1, -8, -18	collagen type I, II, III, IV, V, IX, X, XI, gelatin, laminin
MMP-14	MT1-MMP	MMP-15, -16, -17, -24, -25 (membrane-type MMPs)	collagen type I, II, III, gelatin, laminin, aggrecans, proMMP-2, proMMP-13
MMP-15	MT2-MMP	MMP-14, -16, -17, -24, -25 (membrane-type MMPs)	collagen type I, II, III, gelatin, proMMP-13
MMP-16	MT3-MMP	MMP-14, -15, -17, -24, -25 (membrane-type MMPs)	collagen type I, II, laminin, proMMP-2, proMMP-13
MMP-17	MT4-MMP	MMP-14, -15, -16, -24, -25 (membrane-type MMPs)	fibronectin, fibrin, gelatin
MMP-18	collagenase 4, *Xenopus*	MMP- 1, -8, -13	-
MMP-19	RASI 1	-	collagen type I, IV, gelatin, fibronectin, laminin, aggrecan, entactin, tenascin
MMP-20	enamelysin	-	amelogenin, aggrecans
MMP-21	XMMP	-	-
MMP-22	CMMP	-	gelatin
MMP-23	CA-MMP	-	gelatin
MMP-24	MT5-MMP	MMP-14, -15, -16, -17, -25 (membrane-type MMPs)	proMMP-2, proMMP-13
MMP-25	MT6-MMP	MMP-14, -15, -16, -17, -24 (membrane-type MMPs)	proMMP-2
MMP-26	matrilysin 2	MMP-7	collagen type IV, gelatin, fibrinogen, fibronectin, vitronectin, casein, pro-MMP-9
MMP-27	MMP-22, C-MMP	-	-
MMP-28	epilysin	-	casein

## Data Availability

Not applicable.
